# Freiburg Neuropathology Case Conference: Intraventricular Mass Lesion in a Child

**DOI:** 10.1007/s00062-020-00885-3

**Published:** 2020-02-26

**Authors:** C. A. Taschner, D. Erny, O. Schnell, H. Urbach, I. E. Duman, M. Prinz

**Affiliations:** 1 Department of Neuroradiology, Medical Center – University of Freiburg, Breisacher Straße 64, 79106 Freiburg, Germany; 2 Department of Neuropathology, Medical Center – University of Freiburg, Freiburg, Germany; 3 Department of Neurosurgery, Medical Center – University of Freiburg, Freiburg, Germany

**Keywords:** Choroid plexus tumor, Subependymal giant cell astrocytoma, Supratentorial ependymoma, Central neurocytoma, Pilocytic astrocytoma

## Case Report

An 11-year-old girl presented with increasing headaches that had started approximately 4 weeks earlier. These headaches of high intensity occurred on a daily basis and were predominantly located in the left frontal region. The patient reported an episode of severe pain at night, with a state of disorientation and limited motor control that had first occurred 2 weeks prior to admission. An electroencephalography (EEG) was performed at that time and did not reveal any pathological findings. Neither nausea nor vomiting occurred at any time.

**Fig. 1:**
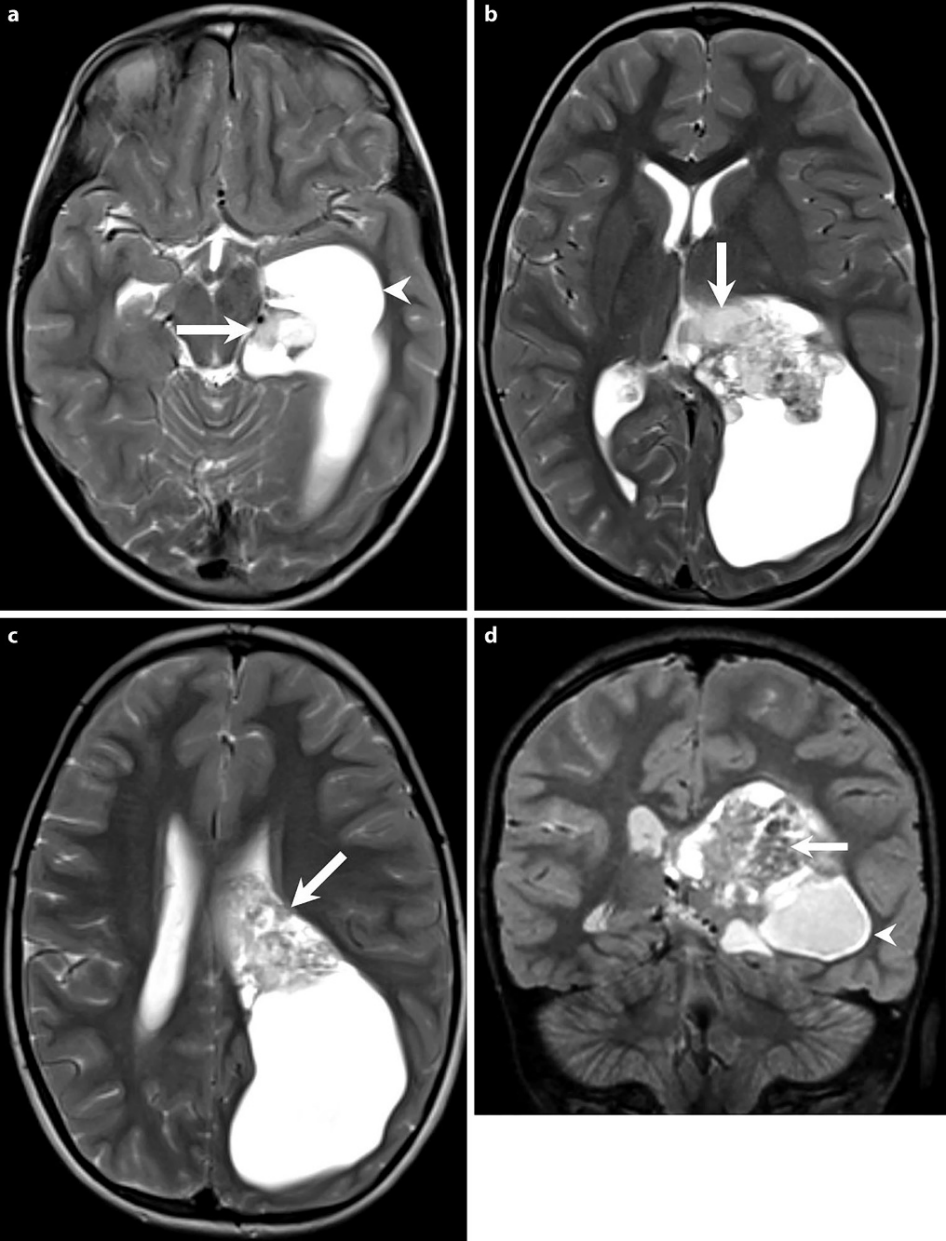
Axial (**a**–**c**) and coronal (**d**) T2-weighted images show an intraventricular tumor extending from the temporal horn through to the cella media of the left lateral ventricle (**a**–**d**, *arrow*). The tumor presents heterogeneous signal intensities with isointense portions next to smaller cystic components. The tumor leads to subsequent dilatation of the left lateral ventricle (**a**, **d**, *arrow head*)

**Fig. 2:**
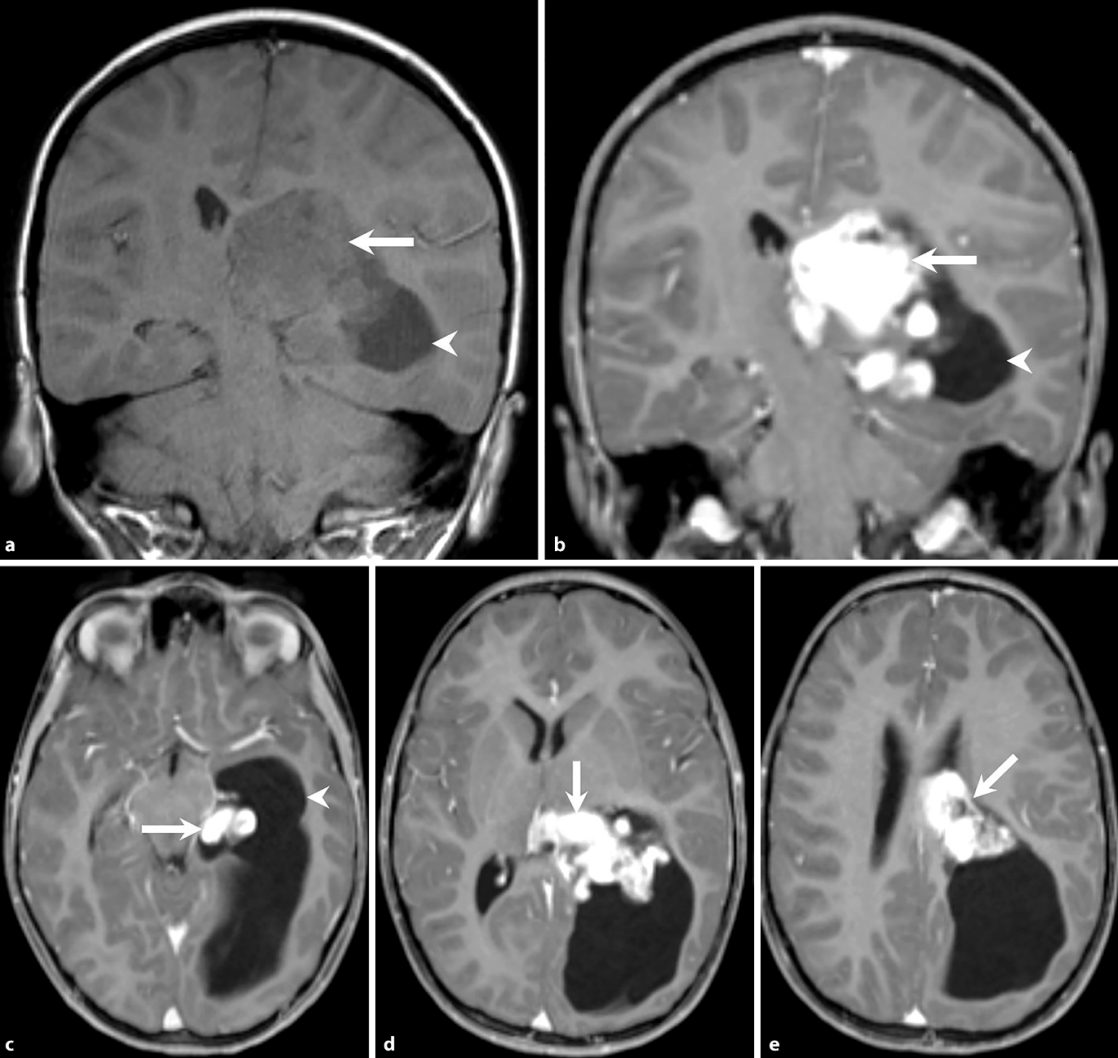
On coronal T1-weighted non-contrast images (**a**) the lesion has a relatively homogeneous appearance and seems isointense compared to the grey matter (**a***arrow*). On coronal (**b**) and axial (**c**–**e**) T1-weighted images obtained after i.v. administration of gadolinium the lesion reveals marked and homogeneous contrast enhancement (*arrow*). The dilatation of the left lateral ventricle is again clearly visualized (**a**–**c***arrowhead*)

**Fig. 3:**
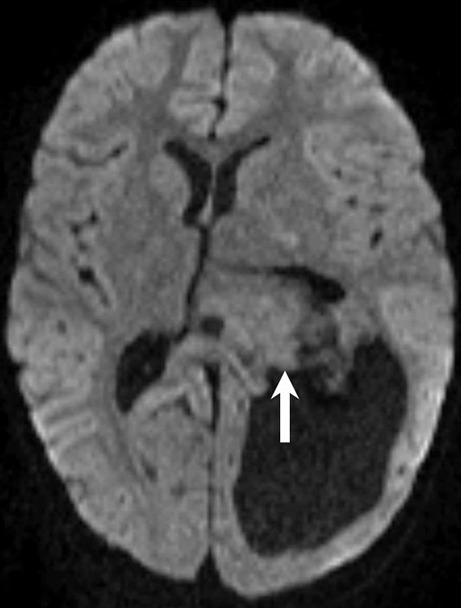
On diffusion-weighted b1000 images the lesion (*arrow*) displays no signs of restricted diffusion making the presence of a tumor with elevated cellularity and high proliferative activity less likely

On admission the clinically and neurological examinations were strictly normal. Brain magnetic resonance imaging (MRI) showed an intraventricular mass lesion (Figs. 1, 2 and 3). The indications for tumor resection were established at the weekly interdisciplinary tumor board meeting.

The operation was performed applying intraoperative neuronavigation, intraoperative ultrasound and intraoperative neuromonitoring. Despite an initially unremarkable course of surgery, the first operation for tumor resection had to be aborted shortly after dural opening due to a newly occurring epidural hematoma. Postoperatively a new visual field loss occurred; otherwise no new neurological deficit was encountered. The second operation for tumor removal was performed 8 weeks later. After opening of the dura a soft predominantly greyish tumor with a sharp border to the surrounding ependymal lining, especially in the direction of the cella media of the left lateral ventricle was developed. In a rostral direction a sharp border to the thalamus was visible. A larger tumor node was visualized in the direction of the temporal horn of the left ventricle which appeared to be covered by a thin layer of parenchyma. After fenestration of the parenchyma under neuronavigation, the greyish tumor of soft consistency could easily be separated from the surrounding tissue and was removed. Finally, the tumor portion directed to the midline, adjacent to the internal cerebral veins was prepared. Here, the tumor borders seemed less well defined. The consistency of the tissue appeared hardened and was of more diffuse yellowish-greyish appearance. Altered tissue was removed via a cavitronic ultrasonic surgical aspirator (CUSA), under neuronavigation and electrophysiological monitoring. Due to increasingly diffuse tumor infiltration and no indication of clear tumor residues, resection was stopped and wound closure accomplished. The postoperative course was uneventful and the patient was discharged home in good clinical condition.

## Imaging

Axial (Fig. 1a–c) and coronal (Fig. 1d) T2-weighted images showed an intraventricular tumor extending from the temporal horn through to the cella media of the left lateral ventricle (Fig. 1a–d, arrow). The tumor presents heterogeneous signal intensities with isointense portions next to smaller cystic components. The tumor led to subsequent dilatation of the left lateral ventricle (Fig. 1a, d, arrowhead). On coronal T1-weighted non-contrast images (Fig. 2a) the lesion had a relatively homogeneous appearance and seemed isointense compared to the grey matter (Fig. 2a, arrow). On coronal (Fig. 2b) and axial (Fig. 2c–e) T1-weighted images after i.v. administration of Gadolinium the lesion revealed marked and homogeneous contrast enhancement (arrow). The dilatation of the left lateral ventricle was clearly visualized (Fig. 2a–c, arrowhead). On diffusion weighted b1000 images (Fig. 3) the lesion displayed no signs of restricted diffusion (arrow). On head CT obtained after the first operation the lesion did not show any calcification (not shown).

## Differential Diagnosis

### Choroid Plexus Tumor

Choroid plexus tumors (CPT) are rare, highly vascularized neoplasms of the central nervous system (CNS) representing <1% of all brain tumors [1], but 12–20% of brain tumors in the first year of life [2, 3]. Choroid plexus tumors are the most common primary brain tumors in children <1 year old. According to the most recent World Health Organization (WHO) classification, CPT consist of choroid plexus papillomas (CPP, WHO grade I), atypical choroid plexus papillomas (aCPP, WHO grade II), and choroid plexus carcinomas (CPC, WHO grade III) [4]. These tumors occur in the ventricular system with the lateral (50%) and fourth (40%) ventricles being the most common locations, affording these tumors the ability to cause hydrocephalus and/or metastasize through the ventricles. Nausea, vomiting, headache, and obtundation are the most common presenting features most likely related to a rise in intracranial pressure. The typical imaging pattern consists of a usually large lobulated (cauliflower-like) mass within the atrium of the lateral ventricle with a strong enhancement and internal linear and branching vascular flow voids. Computed tomography (CT) shows an intraventricular lobular mass with a periventricular interstitial edema due to ventricular obstruction or overproduction of cerebrospinal fluid (CSF). On magnetic resonance imaging (MRI), choroid plexus tumors show a well-delineated T1-isointense to hypointense lobular mass with a marked and homogeneous enhancement. In addition, occasional cysts, calcifications and small foci of necrosis can be present [5]. Imaging cannot reliably distinguish CPP from aCPP or CPC. Extensive invasion of the brain parenchyma, heterogeneity, early CSF spread suggests CPC.

### Subependymal Giant Cell Astrocytoma

Subependymal giant cell astrocytomas (SGCA, SEGA) are benign (WHO grade I), slowly growing glioneuronal tumors arising close to the foramen of Monro in patients with tuberous sclerosis complex (TSC). The SEGA are believed to arise from a subependymal nodule present in the ventricular wall in patients with tuberous sclerosis [6]. The SEGAs are most common CNS neoplasms in TSC, occurring in up to 15% of patients with TSC, whilst rare in absence of TSC. They are principally diagnosed in patients under 20 years of age and can only occasionally be found in older individuals. The SEGAs are often asymptomatic. When symptoms occur they are usually a result of obstructive hydrocephalus due to mass effect. On MRI, SEGAs typically appear as large tumor masses in the vicinity of the foramen of Monro, hypointense to isointense on T1-weighted images with marked contrast enhancement and CT imaging shows an intraventricular, in most cases calcified mass of variable mixed density. Intratumoral hemorrhage is rare [7]. In the absence of TSC in our patient, the diagnosis SEGA seemed less likely.

### Supratentorial Ependymoma

Ependymomas are glial tumors with ependymal differentiation which tend to arise within or abutting to the ventricular system of the brain or central canal of the spinal cord [8]. They are the third most common intracranial neoplasm in children presenting most commonly with seizures. Despite the high incidence of infratentorial ependymomas, supratentorial ependymomas account only for one third of all ependymomas and up to half of these are intraparenchymal. They are rarely seen in the lateral ventricles. They are molecularly distinct tumors and classified as a subtype of ependymomas, which in turn have different epidemiology and prognosis. The two main subgroups of supratentorial ependymomas are characterized by fusions on chromosome 11 involving the coactivator RELA (nuclear factor NF-kappa-B p65 subunit) (affecting older children) or less frequently YAP1 fusions (affecting children <3 years of age) [9]. The incidence of these tumors is bimodal, with one peak in children 1–5 years of age and a smaller peak during the 2–3 decades of life. The CT images show a large mixed isodense/hypodense mass with multiple cysts, fluid-fluid levels, calcifications, significant mass effect, and peritumoral edema. The tumor shows a moderately intense enhancement (solid parts) with necrotic foci. The MRI findings of supratentorial ependymomas are similar to those for intraparenchymal lesions. They are isointense to slightly hypointense in T1-weighted images, and hypointense to hyperintense (high cellularity, calcifications) on T2-weighted images and show a moderate contrast enhancement with necrotic foci [8]. The tumor in our patient lacked calcification, apart from that it seemed a valid differential diagnosis.

### Central Neurocytoma

Central neurocytomas are WHO grade II neuroepithelial intraventricular tumors occurring in young patients (70% diagnosed between 20 and 40 years of age) and account for less than 1% (0.25–0.5%) of intracranial tumors [8]. Headache, increased intracranial pressure, mental status changes and seizures are the most common presenting features of central neurocytomas due to the hydrocephalus secondary to obstruction of the foramen of Monro. Complete surgical resection is often curative for these tumors. The vast majority of central neurocytomas are located within the lateral ventricles around the foramen of Monro. On imaging these tumors appear as heterogeneous masses of variable size and enhancement within the lateral ventricle, typically attached to the septum pellucidum. On CT they appear as mixed solid and cystic (isodense/hyperdense), in most cases calcified, intraventricular, lobular masses with a periventricular interstitial edema due to ventricular obstruction. The MRI shows a characteristic “bubbly” multilobulated appearance with T1-weighted heterogeneous hypointense and T2-weighted hyperintense signal intensities. The T2*/SWI (susceptibility weighted imaging) is useful to visualize calcifications which may be present in up to 50–70% of cases. After gadolinium injection, moderate to strong enhancement is seen [8]. The imaging features of the intraventricular tumor in our patient are in line with the diagnosis of a central neurocytoma, yet they seem to be rare in children.

### Pilocytic Astrocytoma

Pilocytic astrocytomas are low-grade, relatively well-defined, WHO grade I tumors affecting children and young adults with a peak incidence between 5 and 15 years old. They are the most common primary brain tumor of childhood, accounting for 15% of all pediatric brain tumors [9]. A strong association with neurofibromatosis type 1 (NF 1) is known. Pilocytic astrocytomas are most commonly seen in the cerebellum (60%), followed by the optic pathway (25–30%) being the next most common location, particularly in patients with NF1. Other less common locations are the brainstem, cerebral hemispheres particularly in adults, cerebral ventricles, velum interpositum and spinal cord (spinal pilocytic astrocytomas). Clinical presentation varies with location. Headache, nausea and vomiting are the most common symptoms due to the raised intracranial pressure, mainly related to hydrocephalus. Visual symptoms are related with the optic pathway lesions, while ataxia and cerebellar signs are seen in cerebellar lesions. The typical imaging pattern consists of a usually large, cystic mass with a strong enhancing mural nodule. Overall morphology is often determined by cystic component. Computed tomography shows a cystic and solid mass with relatively little peripheral edema. The solid part appears hypodense to isodense in non-enhanced CT compared to gray matter and sometimes shows calcification. after contrast agent administration, they show variable enhancement patterns with variable involvement of cystic components and mural nodules. The MRI shows in 50% of cases a characteristic non-enhancing cyst, strongly enhancing mural nodule appearance with T1-weighted isointense/hypointense and T2-weighted hyperintense solid portions. Solid portions show intense but heterogeneous enhancement after gadolinium injection. Cyst contents appear in T2-weighted images isointense/hyperintense, in fluid-attenuated inversion recovery (FLAIR) images hyperintense to CSF and shows occasionally enhancement [10].

## Histology

### Histology and Immunohistochemistry

**Fig. 4:**
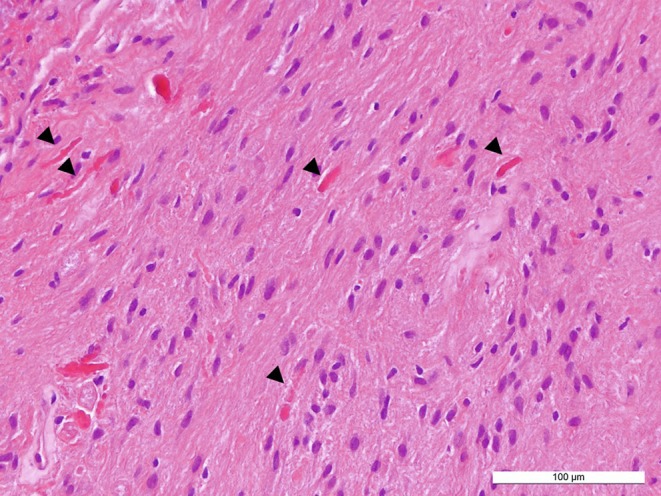
Hematoxylin-eosin stained section showing an astrocytic tumor with Rosenthal fibers (*arrowheads*). Size bar: 100 µm

**Fig. 5:**
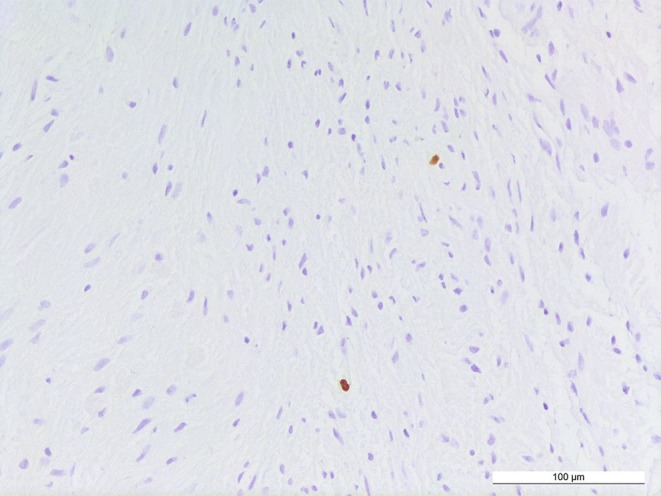
MIB1 staining. The proliferation index is slightly increased tagging less than 5% of tumor cells. Size bar: 100 µm

In the hematoxylin-eosin (H&E) stained sections of the formaldehyde-fixed and paraffin-embedded initial intraoperative biopsy material, a relatively isomorphic neoplasm with slightly increased cellularity without signs of necrosis was observed and classified as pilocytic astrocytoma. The following biopsy material yielded more tumor tissue which exhibited similar histomorphological features: the astrocytic tumor shows predominantly a solid growth pattern, regionally a mucoid matrix and appears to be relatively sharp demarcated from few smaller fragments of the adjacent CNS tissue. The tumor cells display mostly small and round-oval shaped chromatin dense nuclei (Fig. 4). Eosinophilic Rosenthal fibers and protein droplets can be found regionally (Fig. 4). Occasional dystrophic calcifications are seen locally in the present tissue as well as a few and mostly small hemorrhages. Few hemosiderin deposits indicating older bleedings can be detected focally. Mitotic figures are not detectable. Accordingly, the proliferation index is only modestly increased and in total less than 5% of the tumor cells are marked in the Ki67/MIB1 immunohistochemical reaction (Fig. 5). Blood vessels feature partially thickened hyalinized walls. Microvascular proliferation is not present in several regions. Marked desmoplastic components are absent in the present tissue.

**Fig. 6:**
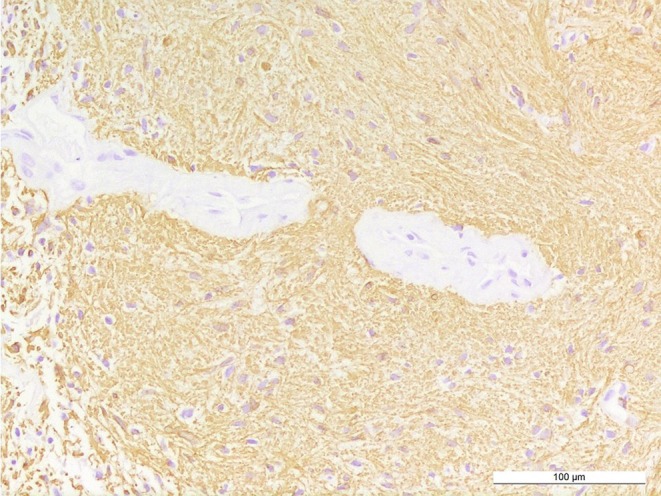
Tumor cells and a GFAP-rich tumor matrix are visualized by immunohistochemical reaction against GFAP. Size bar: 100 µm

**Fig. 7:**
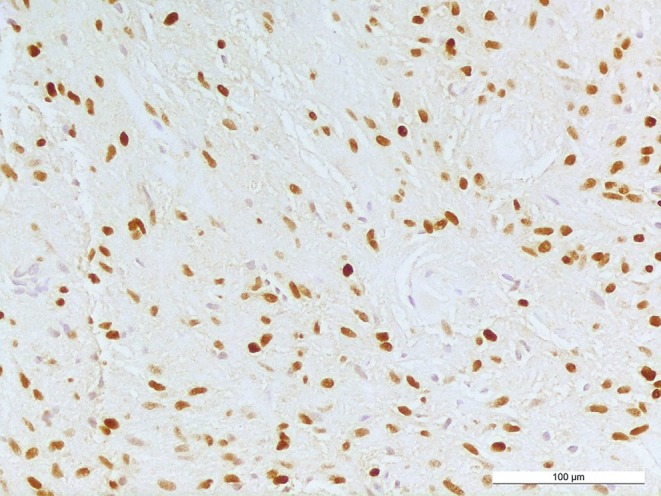
Immunohistochemical staining against OLIG2 reveals positivity in various tumor cells. Size bar: 100 µm

**Fig. 8:**
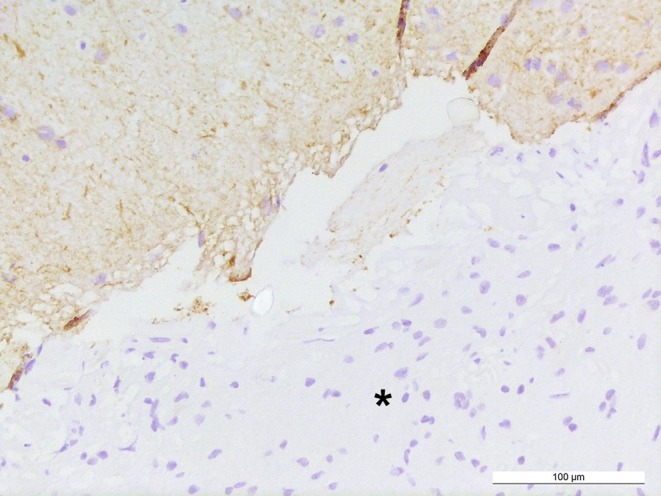
The immunohistochemical reaction against neurofilament remains negative within the tumor tissue (*asterisk*) and shows a positive reaction in sharply demarcated adjacent gliotic CNS tissue. Size bar: 100 µm

Staining for GFAP (glial fibrillary acidic protein) is predominantly positive in the tumor tissue (Fig. 6). Tumor cells show OLIG2 expression, indicative among others for gliomas, including pilocytic astrocytoma and high-grade astrocytoma or oligodendroglioma (Fig. 7). The immunohistochemical reaction against neurofilament confirms absence of neuronal structures within the tumor tissue and highlights a relatively sharp border to adjacent gliotic CNS tissue (Fig. 8). Staining using a mutation-specific (R132H) antibody against isocitrate dehydrogenase 1 (IDH1), indicative for IDH-mutated astrocytomas and oligodendrogliomas [11], revealed no specific reaction in the present tumor (not shown). The nuclear expression of the transcriptional regulator ATRX is retained in the tumor cells (not shown).

In summary, the histopathological finding of a glial, isomorphic, relatively well-demarcated tumor with slightly elevated cellularity exhibiting characteristic Rosenthal fibers together with low proliferative activity as well as absence of microvascular proliferation and necrosis leads consequently to the diagnosis of pilocytic astrocytoma, WHO grade I. Signs of anaplastic transformation are not found. The diagnosis is further supported by detection of the BRAF-KIAA1549 fusion that constitutively activates the MAPK pathway and occurs in approx. 70% of pilocytic astrocytomas [12, 13]. The diagnosis was independently confirmed by the brain tumor reference center in Berlin (Germany).

Differential diagnoses for the current tumor include primarily tumors usually found in the lateral ventricles during childhood, e.g. ependymal tumors (such as ependymoma, WHO grade II), tumors derived from choroid plexus (such as choroid plexus papilloma, WHO grade I) or central neurocytoma, WHO grade II. The SEGAs typically appear in the wall of lateral ventricles and are associated with the tuberous sclerosis syndrome and harbor large ganglionic astrocytic cells [13, 14]. As another differential diagnosis diffuse gliomas can be considered since they may be located predominantly in the ventricle system [13].

Concerning these differential diagnoses, perivascular pseudorosettes a common feature of ependymoma are not present in the specimen. Furthermore, OLIG2 expression is present in the tumor cells and described to be absent in ependymal tumors [15]. The morphological features of choroid plexus tumors are quite distinct with a papillary pattern composed of a single layer of epithelial tumor cells. In contrast, the present tumor displays mainly a solid growth pattern with monomorphic glial tumor cells. Additionally, expression of neuronal markers is absent in the present specimen and oppose the diagnosis of tumors with an at least partial neuronal differentiation like central neurocytoma and SEGA. The differential diagnoses of diffuse gliomas including the highly malignant pediatric tumor diffuse midline glioma are ruled out by a lack of histomorphological hallmarks including increased mitotic rate, diffuse infiltration. Furthermore, H3 K27M mutation and mutations in the IDH1 and IDH2 genes are absent. Pilomyxoid astrocytomas are a more rare variant of pilocytic astrocytoma [16]. In contrast to the present tumor they are histologically characterized by an angiocentric composition of the tumor cells and prominent myxoid alterations.

## Diagnosis

### Intraventricular Pilocytic Astrocytoma (WHO I)

Intraventricular tumors are relatively symptomless until they enlarge and obstruct the pathways of CSF, producing obstructing hydrocephalus and leading to an increased intracranial pressure [17]. Pilocytic astrocytomas are the most common glioma present in children and young adults and composed of neoplastic glial cells [18]. They are circumscribed, benign, slowly growing and mainly located in infratentorial regions and cerebral midline structures. Tumors localized in the lateral ventricles of the cerebral hemispheres account for less than 1% of all intracranial tumors but are relatively more frequent in the pediatric population [17]. Pilocytic astrocytomas rarely present as intraventricular tumors and should be included in the differential diagnosis in a pediatric population. The intraventricular location of a glial tumor leads to the assumption that the present tumor initially originated from the brain parenchyma close to the ventricle system and subsequently grew into the ventricles [13].
